# The impact of energy poverty on the health capital of middle-aged and older adult residents in rural China

**DOI:** 10.3389/fpubh.2025.1580069

**Published:** 2025-04-11

**Authors:** Cuiting Yu, Tianrun Li, Qin Wan

**Affiliations:** School of Economics and Management, Southwest Petroleum University, Chengdu, China

**Keywords:** energy poverty, health capital, rural China, aging population, ordered logit model

## Abstract

**Introduction:**

Energy poverty significantly affects the well-being of vulnerable populations, particularly middle-aged and older adult individuals in rural China. This study investigates how energy poverty impacts health capital, using data from the China Health and Retirement Longitudinal Study (CHARLS).

**Methods:**

We employed the Multidimensional Energy Poverty Index (MEPI) to measure energy poverty and used an ordered logit model to analyze its effects on self-rated health (SRH) as a proxy for health capital. The study utilized longitudinal data from 2013, 2015, 2018, and 2020, covering 9,464 observations, and included control variables such as age, gender, and chronic disease status. Endogeneity was addressed using instrumental variables and propensity score matching.

**Results:**

The findings indicate that energy poverty has a significant negative impact on health capital, with a regression coefficient of −0.221(p<0.01), lowering self-rated health levels. This effect is consistent across physical health, mental health, and daily functioning. Heterogeneity analysis reveals that individuals with lower education levels and those in southern rural areas experience more severe health impacts. Mediation tests confirm that indoor environmental conditions partially mediate this relationship.

**Discussion:**

The study underscores the urgent need for targeted interventions to mitigate energy poverty in rural China. Expanding access to clean energy, improving rural infrastructure, and providing financial subsidies are critical. Education and regional policies should also be prioritized to address disparities.

## 1 Introduction

Energy serves as the foundation of modern society, underpinning economic growth, technological advancement, and overall human wellbeing. However, more than 700 million people worldwide still live without access to electricity, while billions rely on inefficient and highly polluting energy sources, such as biomass fuels and coal, for daily activities (1). The lack of adequate energy access or its improper use not only perpetuates the cycle of poverty but also poses significant threats to human health (2–4). In many developing regions, limited access to reliable and affordable energy sources constrains economic opportunities, restricts access to education and healthcare, and increases reliance on hazardous traditional fuels. As a result, energy poverty has emerged as a critical issue that requires urgent attention (5). The United Nations Development Programme (UNDP) defines energy poverty as an absence of sufficient choice in accessing adequate, affordable, reliable, high-quality, and environmentally and health-friendly energy services to support economic development opportunities for communities (6). According to the International Energy Agency (IEA), energy poverty refers to a lack of access to electricity and dependence on traditional biomass for cooking and heating. Similarly, the Asian Development Bank (ADB) defines energy poverty as the inability to cook with modern fuels and the lack of minimum energy required to meet basic needs and income-generating activities after sunset (7). Many scholars, when studying energy poverty, incorporate Amartya Sen’s Capability Approach, framing energy poverty as a lack of capability or freedom to meet basic energy needs (8–11). This theory emphasizes that poverty is not merely a shortage of resources but rather a limitation on people’s ability to achieve fundamental functions and lead a dignified life. In the context of energy poverty, this perspective extends beyond mere energy availability to examine whether individuals or communities have the capacity to access, afford, and efficiently utilize energy to meet basic survival needs and improve their quality of life. In China, energy poverty is particularly prominent in rural areas. Consequently, Chinese scholars have actively investigated this phenomenon, conducting a series of studies. For example, Liao et al. (12) argue that due to factors such as income levels and energy availability, energy-poor households primarily rely on solid fuels for cooking and heating. Yang and Zhang (13) further assert that the root cause of energy poverty is not the inaccessibility or unaffordability of energy services but rather the deprivation of people’s capability to utilize them effectively.

Various methods have been proposed to effectively measure energy poverty. At the household level, these methods can be broadly categorized into two main types: unidimensional measurement methods and multidimensional measurement methods (14). Unidimensional measurement methods typically assess the extent of energy poverty based on a single indicator, such as energy consumption (15) or energy expenditure (16). In contrast, multidimensional measurement methods take into account multiple dimensions related to energy poverty, offering a more comprehensive evaluative perspective (17). The Multidimensional Energy Poverty Index (MEPI), developed by Nussbaumer et al. (9) based on the Oxford Poverty and Human Development Initiative’s Multidimensional Poverty Index (MPI), provides a more nuanced representation of the complexity and diversity of energy poverty. As a result, it has been widely adopted in academic research. For instance, Sadath and Acharya (8) applied MEPI to assess energy poverty in India, offering empirical insights for optimizing energy policies in developing countries. Similarly, Mendoza et al. (18) utilized the MEPI approach to examine energy poverty in the Philippines, while Sokołowski et al. (19) employed MEPI to measure household energy poverty in Poland. Many scholars have also explored energy poverty in China using this method. For example, He et al. (20), Xie (21), Zhao et al. (22), and Wang and Lin (23) have applied the MEPI to measure energy poverty in China. Given MEPI’s strong applicability and its ability to provide a reliable analytical framework, this study adopts MEPI as the core measurement tool to analyze energy poverty in rural China.

Energy poverty is closely related to health (24). In regions with limited energy access, many households still rely on solid fuels such as wood, coal, and animal dung as their primary energy sources. The combustion of these fuels in poorly ventilated environments leads to indoor air pollution, which adversely affects individual health (25). A wide consensus has developed that energy poverty affects a nation’s health (26–28), contributing to a range of negative health outcomes. As one of the largest developing countries in the world, China also faces an energy poverty crisis in some households. The impact of energy poverty on health has been supported by empirical data from China (29–31), including some studies specifically focusing on rural areas. For details, see Table 1. However, most of these studies have not focused on specific demographic groups. The health of middle-aged and older adult residents in rural China is significantly affected by energy poverty. On one hand, they have higher health demands; however, their low income and limited education create barriers to accessing and utilizing modern energy sources. On the other hand, the inadequate energy infrastructure in rural areas often forces residents to rely on traditional fuels for daily needs, further exacerbating health risks. According to a study, the likelihood of a home suffering from energy poverty increases by 2.4 percentage points when it is located in a rural area (32). Although existing research has made some progress in examining energy poverty and its health impacts, studies on the relationship between health capital and energy poverty among middle-aged and older adult populations in rural China remain insufficient. Given that this demographic constitutes a vital segment of rural communities and that their health capital is potentially threatened by energy poverty, greater attention is needed. This study, therefore, focuses on the health capital of middle-aged and older adult individuals in rural China. By thoroughly exploring the impact of energy poverty on their health capital, this research seeks to provide theoretical support for the development of more targeted energy and health policies, ultimately contributing to the enhancement of overall health and quality of life in rural areas.

**Table 1:** Related literature.

Author	Scope	Data	Conclusion
Pan et al. (24)	Global	Annual data for a broad panel of 175 countries over the period 2000–2018	This study confirms that energy poverty has a significant adverse impact on public health and reveals the moderating role of living standards in this relationship.
Oum (27)	Lao	Lao Economic Consumption Survey (LECSs)	Energy poverty primarily affects low-income households and negatively impacts their average years of education and health status.
Banerjee et al. (28)	Developing countries	Annual data for a broad panel of 50 countries over the period 1990–2017	There is a greater effect of energy development index on life expectancy rates where the poverty headcount ratio is high. Conversely, energy development index has a greater effect on infant mortality rates where the poverty headcount ratio is low, or income per capita is high.
Zhang et al. (29)	China	Chinese General Social Survey (CGSS)	The health of residents in less developed provinces is more severely affected, and multidimensional energy poverty deteriorates the physical health of rural residents but impacts the mental health of urban residents.
Xu et al. (30)	China	China Family Panel Studies (CFPS)	Energy poverty significantly affects residents’ health and indirectly exacerbates its negative effects through pathways such as resource accessibility, opportunities for physical exercise, and medical expenditures. Moreover, its impact is moderated by factors such as age and household income.
Zhang et al. (31)	Rural China	China Family Panel Studies (CFPS)	Energy poverty significantly exacerbates the depression levels of individuals with high depression, but it has no significant effect on individuals with moderate or low depression.

## 2 Theoretical basis

### 2.1 Grossman health demand model

**Figure 1:**
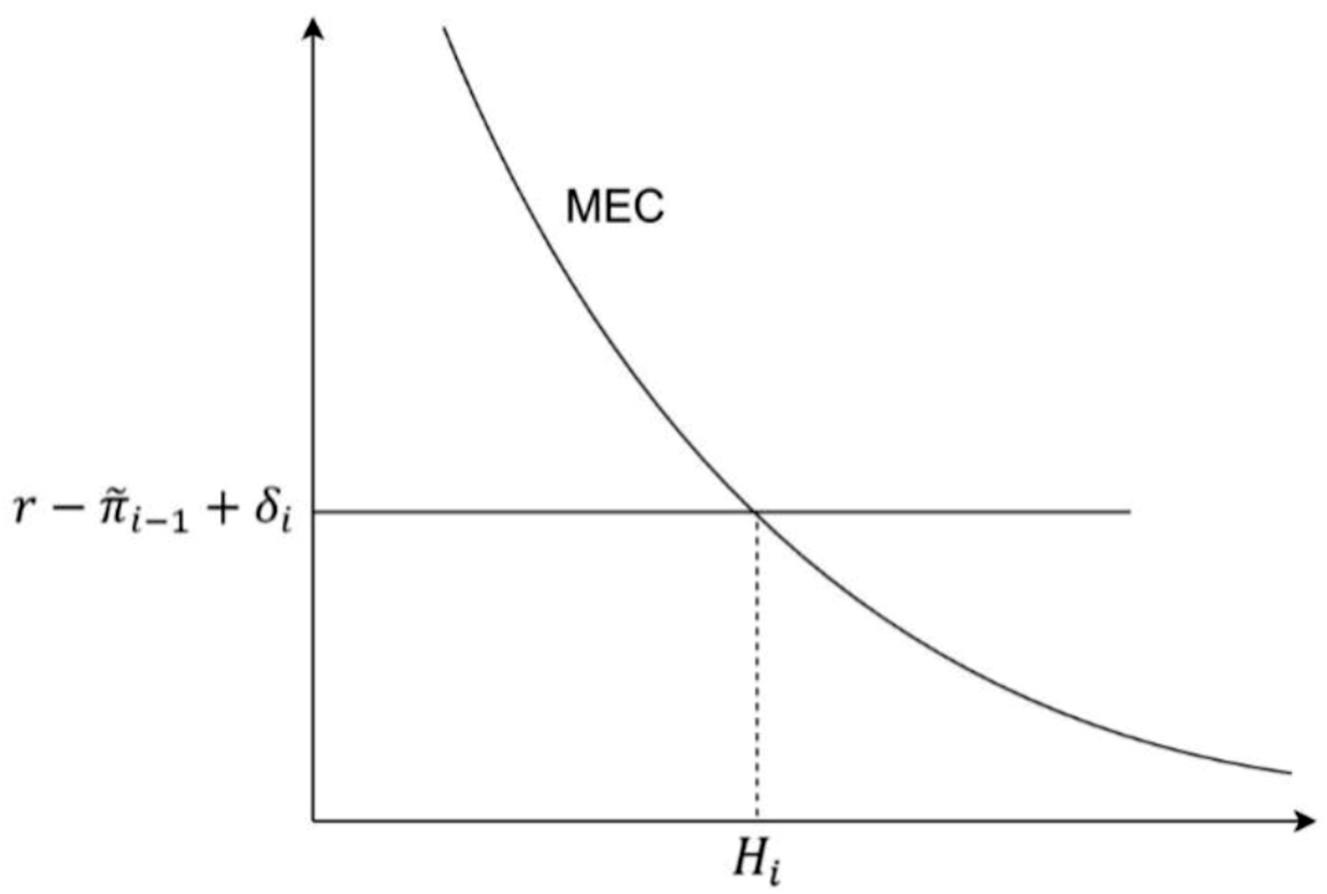
The demand curve for health capital and the equilibrium point.

The Grossman health demand model is a classic theoretical framework in health economics that conceptualizes health as a consumption good, a factor of production, and a form of capital. In this model, individuals actively manage their own health, exercising subjective agency (33). Moreover, the model provides a strong explanatory framework for various health-related phenomena, including the relationship between socioeconomic status and health. According to the Grossman model, the following equation can be derived:


(1) $$\gamma_{i}+\alpha_{i}=r-{\tilde{\pi }}_{i-1}+\delta_{i}$$

where *i* represents age, *γ_i_* denotes the return on health as a productive input, and *α_i_* represents the return on health as a consumption good. Due to diminishing marginal returns and diminishing marginal utility, the marginal efficiency curve (MEC) of health capital slopes downward (Figure 1). Investing in health requires individuals to forgo the opportunity to allocate resources to other market investment projects, whose returns are equivalent to the interest rate *r* and the depreciation rate of health *δ_i_*. Considering the rate of change in marginal cost across different periods ${\tilde{\pi }}_{i-1}$ (representing the change in marginal cost from period *i* − 1 to *i*), the return on health must be at least $r-{\tilde{\pi }}_{i-1}+\delta_{i}$ to ensure that health investment remains competitive with alternative investments. When Equation 1 holds, it signifies that the marginal return on health investment equals the cost of utilizing health capital. And the sum of the returns from alternative investments, the rate of change in marginal costs, and the depreciation rate ( $r-{\tilde{\pi }}_{i-1}+\delta_{i}$ ) represents the actual price of health capital.

### 2.2 Energy poverty and the expansion of the health demand model

For households, one significant way in which energy poverty affects health is through indoor pollution caused by the use of non-clean energy, which in turn negatively impacts health. Since pollution physically affects human health, it exogenously alters an individual’s health by accelerating health depreciation (34). The impact of energy poverty on health is similar to that of environmental pollution, as both affect physical health. Consequently, energy poverty also influences an individual’s health level by affecting health depreciation *δ_i_*. The health depreciation rate can be expressed as:


(2) $$\delta_{i}=\delta_{0}e^{\tilde{\delta }i}P_{i}^{\psi }S_{i}^{\Phi }$$

where *δ*_0_ represents the initial health depreciation rate; *i* denotes age, which affects the health depreciation rate with a constant elasticity $\tilde{\delta }$ ; *P_i_* represents the state of pollution; and *S_i_* represents other variables that influence health. Equation 2 helps to understand the mechanism through which energy poverty impacts health capital. Energy poverty leads to indoor pollution, which directly affects the depreciation of health capital.

Since *γ_i_* and *α_i_* share similar properties, to simplify the model and facilitate analysis, it is assumed that acquiring more healthy time is the sole motivation for investing in health. This assumption leads to the following equation:


(3) $$\gamma_{i}=r-{\tilde{\pi }}_{i-1}+\delta_{i}$$

Taking the partial derivative of Equation 3 with respect to *i* yields the rate of decline in an individual’s health level over their lifetime: ${\tilde{H}}_{i}=-s_{i}\varepsilon_{i}{\tilde{\delta }}_{i}$ , where the tilde represents the rate of change of a variable over time. Here, $s_{i}=\frac{\delta_{i}}{r-{\tilde{\pi }}_{i-1}+\delta_{i}}$ , denotes the proportion of health depreciation in the total cost of health capital, while $\varepsilon_{i}=-\frac{\partial \ln H_{i}}{\partial \ln\left(r-{\tilde{\pi }}_{i-1}+\delta_{i}\right)}=-\frac{\partial \ln H_{i}}{\partial \ln\gamma_{i}}=-\frac{\partial \ln H_{i}}{\partial \ln G_{i}}$ , represents the elasticity of health level with respect to health costs.

Differentiating Equation 3 with respect to $\ln\delta_{i}$ results in:


(4) $$\frac{dlnH_{i}}{dln\delta_{i}}=-s_{i}\varepsilon_{i}<0$$

Equation 4 indicates that when health depreciation changes due to energy poverty, an individual’s health also changes in the opposite direction. In addition to affecting health changes, energy poverty also impacts variations in health investment. By definition, net investment in the stock of health equals gross investment minus depreciation: $H_{i+1}-H_{i}=I_{i}-\delta_{i}H_{i}$ , where *I_i_* represents gross health investment. Rewriting this equation yields:


(5) $$\ln I_{i}=\ln H_{i}+\ln\left({\tilde{H}}_{i}+\delta_{i}\right)$$

Taking the derivative of Equation 5 with respect to $\ln\delta_{i}$ , we obtain:


(6) $$\frac{dlnI_{i}}{dln\delta_{i}}=\frac{\left(1-s_{i}\varepsilon_{i}\right)\left(\delta_{i}-s_{i}\varepsilon_{i}\tilde{\delta }\right)+{s_{i}}^{2}\varepsilon_{i}{\tilde{\delta }}_{i}}{\delta_{i}-s_{i}\varepsilon_{i}{\tilde{\delta }}_{i}}>0$$

**Figure 2:**
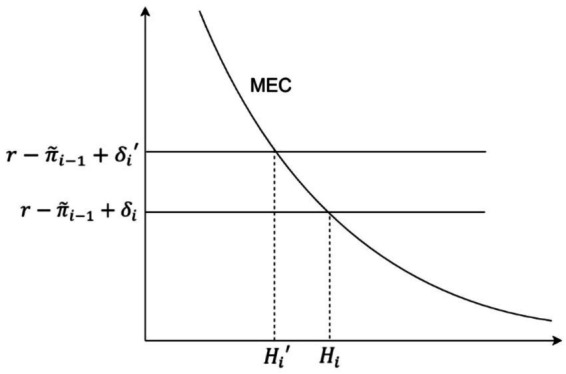
The negative health impacts of energy poverty.

Equation 6 demonstrates that energy poverty leads individuals to increase their health investments. However, this increased health investment often fails to restore their health levels to those of individuals who are not experiencing energy poverty. Individuals in energy poverty allocate part of their resources to offset the utility loss caused by energy poverty. To maximize utility, they do not invest sufficient resources to match the health levels of those not in energy poverty, ultimately sacrificing a certain degree of health. This dynamic can be better understood graphically: when energy poverty increases health depreciation from $\delta_{i}$ to ${\delta_{i}}^{\prime }$ , the rising cost of health capital causes health levels to decline from $H_{i}$ to ${H_{i}}^{\prime }$ (Figure 2). To empirically test the theoretical effect of energy poverty on health capital, the following research hypothesis is proposed:

Hypothesis 1: Energy poverty has a negative impact on residents’ health capital.

Hypothesis 2: Energy poverty affects residents’ health capital by deteriorating indoor environmental conditions.

### 2.3 Heterogeneity analysis of the health effects of energy poverty

The previous analysis is based on the assumption that all groups share common characteristics. However, health disparities exist widely, and the impact of energy poverty on health varies among individuals. For instance, according to the Efficient Producer Hypothesis, health disparities arise because, compared to less-educated individuals, those with higher education levels are more efficient in producing health (35). Education enhances an individual’s willingness to invest in long-term assets, including health. Additionally, more educated individuals are better at following medical advice and adhering to complex treatment regimens. To model this phenomenon, assume the gross health investment production function is given by: $I_{i}=I_{i}\left(M_{i},TH_{i};E_{i}\right)$ , where the function is homogeneous of degree one and can be rewritten as:


$$I_{i}=M_{i}g\left(t_{i};E_{i}\right)$$

where $t_{i}=TH_{i}/M_{i}$ , *M_i_* represents medical services received in period *i*, and *TH_i_* represents the time spent on health-promoting activities, such as exercise. The marginal product of time and medical services in health investment are given by: $\frac{\partial I_{i}}{\partial TH_{i}}=\frac{\partial g}{\partial t_{i}}=g^{\prime }$ , $\frac{\partial I_{i}}{\partial M_{i}}=g-t_{i}g^{\prime }$ . In the gross health investment function, the marginal product of human capital (education) *E_i_* is:


$$\frac{\partial I_{i}}{\partial E_{i}}=M_{i}\frac{\partial \left(g-t_{i}g^{\prime }\right)}{\partial E_{i}}+TH_{i}\frac{\partial g^{\prime }}{\partial E_{i}}$$

If a circumflex over a variable denotes a percentage change per unit change in *E*, the last equation can be rewritten as:


$$r_{H}=\frac{\partial I_{i}}{\partial E_{i}}\frac{1}{I_{i}}=\left[\frac{M_{i}\left(g-t_{i}g^{\prime }\right)}{I_{i}}\right]\left[\frac{g\hat{g}-t_{i}g^{\prime }{\hat{g}}^{\prime }}{g-t_{i}g^{\prime }}\right]+\left[\frac{TH_{i}g^{\prime }}{I_{i}}\right]\left[{\hat{g}}^{\prime }\right]$$

This equation implies that a unit increase in human capital leads to a weighted average change in total health investment based on the marginal products of time and medical services. Since human capital enhances productivity, $r_{H}>0$ . Education increases the marginal productivity of direct inputs, which in turn reduces the amount of these inputs needed to produce a certain level of gross investment. As a result, without any change in input prices, an increase in education leads to a reduction in average or marginal costs. If $\hat{\pi }$ represents the percentage change in average or marginal cost, $\hat{\pi }=-r_{H}$ . As a result, differences in health costs lead to variations in health levels.

When facing energy poverty, individuals with different levels of human capital exhibit different responses, leading to varied health impacts. The relationship between the elasticity of the marginal efficiency of capital (MEC) curve and human capital *E* confirms this:


$$\frac{\partial \varepsilon_{i}}{\partial E_{i}}=\frac{\partial \varepsilon_{i}}{\partial \ln H_{i}}\frac{\partial \ln H_{i}}{\partial \ln I_{i}}\frac{\partial \ln I_{i}}{\partial E_{i}}=-\frac{r_{H}s_{i}\left(\delta_{i}+{\tilde{H}}_{i}\right)}{\delta_{i}\left(1+s_{i}\varepsilon_{i}\right)}$$

**Figure 3:**
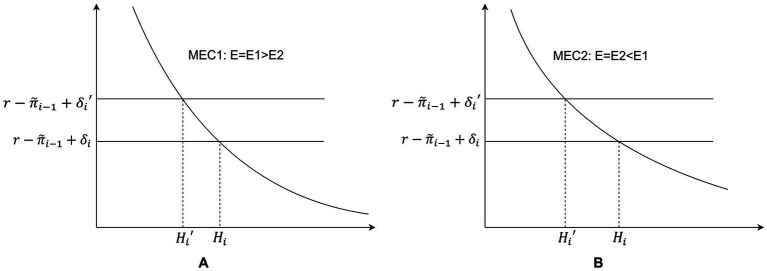
**(A)** Changes in health capital stock in individuals with higher human capital in the face of energy poverty. **(B)** Changes in health capital stock in individuals with lower human capital in the face of energy poverty.

Since $I_{i}>0$ , it follows that $\delta_{i}+{\tilde{H}}_{i}>0$ , leading to $\frac{\partial \varepsilon_{i}}{\partial E_{i}}<0$ . This indicates that individuals with higher human capital experience a lower impact from external factors on their health compared to those with lower human capital. When faced with the threat of energy poverty, individuals seek to maximize utility by increasing their investment ( $\frac{dlnI_{i}}{dln\delta_{i}}>0$ ) to maintain an appropriate level of marginal health output. However, the efficiency of health investment varies among individuals with different levels of human capital ( $r_{H}>0$ ). Consequently, the changes in health status due to external factors also differ. In graphical representation, individuals with different levels of human capital exhibit varying elasticities ( $\frac{\partial \varepsilon_{i}}{\partial E_{i}}<0$ ), leading to heterogeneous effects of energy poverty on their health status (Figure 3).

Similarly, regional differences may also result in variations in health capital levels among individuals affected by energy poverty. Given China’s vast territory and significant temperature differences between the north and south, energy demands vary accordingly. In northern China, where cold weather necessitates heating, energy poverty is likely to have a more severe impact on residents’ health. To examine whether energy poverty leads to heterogeneous effects on health capital, the following research hypothesis is proposed:

Hypothesis 3: Energy poverty has heterogeneous effects on residents’ health capital, influenced by both educational attainment and regional differences.

## 3 Materials and methods

### 3.1 Data sources and variables

#### 3.1.1 Data sources

This study utilizes data from the China Health and Retirement Longitudinal Study (CHARLS), a large-scale interdisciplinary survey project jointly conducted by Wuhan University and Peking University. The CHARLS database aims to collect high-quality microdata that are representative of Chinese households and individuals aged 45 and above, providing essential information for analyzing China’s aging population. The CHARLS questionnaire contains a rich set of information, including basic demographic characteristics, family structure and household finances, health status, healthcare utilization and insurance, retirement and pensions, and consumption patterns. This comprehensive dataset fully meets the needs of this study. The study selects longitudinal data from the 2013, 2015, 2018, and 2020 waves of CHARLS. After data cleaning, the final dataset comprises 9,464 observations, covering multiple health-related variables.

#### 3.1.2 Variable selection

Health capital. Following the approach of Zhao Ming et al. (36), this study employs self-rated health (SRH) as a measure of individual health capital. Compared to single indicators such as disease incidence or medical expenditures, SRH more effectively captures middle-aged and older adult individuals’ overall perception of their health status. This subjective assessment is crucial, as it significantly influences life satisfaction, labor participation, and consumption behavior. Although SRH is a self-reported measure, it is highly correlated with various objective health indicators (e.g., chronic diseases, functional ability), reflecting long-term and multifaceted health outcomes beyond specific diseases or symptoms (37–39). The self-rated health variable (*Health*) is an ordinal variable, ranging from 1 (very poor) to 5 (very good), with higher values indicating better perceived health status.

Energy poverty. Gafa et al. (40) refined Nussbaumer’s Multidimensional Energy Poverty Index (MEPI), making it more applicable to rural areas. Based on their framework and incorporating the adaptation approach of Yang and Zhang (13) for the Chinese context, this study selects six indicators to measure energy poverty: access to electricity, access to tap water, access to piped gas, type of cooking fuel used, proportion of income spent on electricity and water bills, proportion of income spent on fuel costs (See Table 2 for details on energy poverty measurement).

**Table 2:** Energy poverty evaluation indicators.

Dimension	Indicators	Cut-offs	Poor if…
Accessibility	Lighting	Use of modern energy	No
	Cooking		No
Convenience	Piped gas	Availability of gas pipeline	No
	Water supply	Availability of tap water	No
Affordability	Share of electricity and water expenditure	>5%	Yes
	Share of fuel expenditure		Yes

Control variables. This study includes sleep duration, presence of chronic diseases, age, consumption level, gender, exercise habits, and hospitalization in the past year as control variables (41, 42). These factors are widely recognized as being closely associated with individual health status (Table 3).

**Table 3:** Variables and their notation.

Variables	Symbol	Description
Dependent variable		
Self-rated health	Health	Ordinal variable (1 = Very poor, 5 = Very good)
Independent variable		
Energy poverty	Energypoverty	Evaluated based on accessibility, convenience, and affordability
Control variables		
Sleep duration	Sleeping	Hours of sleep per day
Chronic disease	Chronic	1 = Yes, 0 = No
Age	Age	Continuous variable
Consumption level	Expenditure	Household expenditure
Gender	Gender	1 = Male, 0 = Female
Exercise habits	Exercise	1 = Yes, 0 = No
Hospitalization (past year)	Hospital	1 = Yes, 0 = No

### 3.2 Methods for measuring energy poverty

Energy poverty, as a composite indicator, requires determining a specific value using comprehensive evaluation methods. This study adopts the entropy method to calculate the index. The detailed steps are as follows:

Step 1: Data standardization. Let $x_{j}^{\text{max}}$ denote the maximum value of the *j*-th indicator across evaluation objects, and $x_{j}^{\text{min}}$ denote the minimum value of the *j*-th indicator. For positive indicators, the standardization formula is:


$$z_{j}=\frac{x_{j}-x_{j}^{\text{min}}}{x_{j}^{\text{max}}-x_{j}^{\text{min}}}$$

For negative indicators, the standardization formula is:


$$z_{j}=\frac{x_{j}^{\text{max}}-x_{j}}{x_{j}^{\text{max}}-x_{j}^{\text{min}}}$$

After standardization and direction adjustment, the value range of *z_j_* is [0, 1].

Step 2: Constructing the normalized matrix. Based on the standardized indicator *z_j_*, the normalized matrix *p_ij_* is constructed as follows:


$$p_{ij}=\frac{z_{ij}}{\overset{n}{\underset{i}{\sum }}z_{ij}}$$

Here, *z_ij_* represents the standardized value of the *j*-th indicator for the *i*-th object, $i\in \left\{1,2,\ldots ,n\right\}$ where *n* is the number of objects, and $j\in \left\{1,2,\ldots ,m\right\}$ where *m* is the total number of indicators.

Step 3: Calculating the entropy value. Based on the normalized matrix *p_ij_*, the entropy value of the *j*-th indicator is calculated as:


$$e_{j}=-k\overset{n}{\underset{i}{\sum }}p_{ij}\ln p_{ij}$$

where *k* is a constant, defined as


$$k=\frac{1}{\ln n}>0.$$

Step 4: Calculating the degree of redundancy in information entropy. Based on the entropy value *e_j_*, the redundancy degree of information entropy is computed as:


$$d_{j}=1-e_{j}$$

Step 5: Calculating the weight of each indicator. Based on the redundancy degree *d_j_*, the weight of the *j*-th indicator relative to its corresponding dimension is calculated as:


$$w_{j}=\frac{d_{j}}{\overset{m_{l}}{\underset{j=1}{\sum }}d_{j}}$$

Here, *l* represents the number of dimensions, and energy poverty includes three dimensions. *m_l_* denotes the number of sub-indicators under each dimension. The weight *w_j_* reflects the contribution of the *j*-th indicator to the composite score. The larger the weight, the greater the indicator’s influence on the composite score. The final computed score ranges between 0 and 1. Based on the research by Nussbaumer et al. (9), this study considers a score below 0.33 as indicating a state of energy poverty.

### 3.3 Construction of the ordered logit regression model

The dependent variable, self-rated health (*Health*), is ordinal in nature, but the exact differences between categories are unknown. Therefore, this study constructs an ordered logit model. A latent variable, Health*, is introduced to establish the relationship between observable explanatory variables and the observed ordinal dependent variable *Health*, which takes values from 0 to *K*. The latent health status $\mathrm{Healt}h_{\mathrm{it}}^{*}$ of individual *i* at time *t* is linearly determined by *Energypoverty_it_*, *Control_it_*, and two unobservable components, *u_i_* and *ε_it_*:


(7) $$\mathrm{Healt}{h_{\mathrm{it}}}^{*}=\mathrm{\alpha Energypovert}y_{\mathrm{it}}+\mathrm{\beta Contro}l_{\mathrm{it}}+u_{i}+\varepsilon_{\mathrm{it}}$$

The mapping between the latent variable $\mathrm{Healt}{h_{\mathrm{it}}}^{*}$ and observed ordinal outcomes $\mathrm{Healt}h_{\mathrm{it}}$ is governed by:


$$\mathrm{Healt}h_{\mathrm{it}}=k\;\mathrm{if}\;\tau_{ik}<\mathrm{Healt}{h_{\mathrm{it}}}^{*}<\tau_{ik+1}\;k=1,\cdots ,K$$

where $\tau_{ik}$ denotes individualized thresholds. Following standard identification constraints, the boundary thresholds are set as $\tau_{i1}=-\infty$ and $\tau_{iK}=\infty$ , with strictly monotonic thresholds satisfying:


$$-\infty <\tau_{ik}<\tau_{ik+1}<\infty ,\forall k=2,\cdots ,K-1$$

The error term $\varepsilon_{\mathrm{it}}$ follows a logistic probability distribution characterized by:


$$\begin{array}{l} F\left(\varepsilon_{\mathrm{it}}|\mathrm{Energypovert}y_{\mathrm{it}},\mathrm{Contro}l_{\mathrm{it}},u_{i}\right)\\ \;=F\left(\varepsilon_{\mathrm{it}}\right)=\frac{1}{1+\exp\left(-\varepsilon_{\mathrm{it}}\right)}\text{≡}\Lambda \left(\varepsilon_{\mathrm{it}}\right) \end{array}$$

The conditional probability of observing health category *k* for individual *i* at time *t* is derived as:


$$\begin{array}{l} \mathrm{Pr}\left(\mathrm{Healt}h_{\mathrm{it}}=k|\mathrm{Energypovert}y_{\mathrm{it}},Control_{\mathrm{it}},u_{i}\right)\\ \;=\Lambda \left(\tau_{ik+1}-\mathrm{\alpha Energypovert}y_{\mathrm{it}}-\mathrm{\beta Contro}l_{\mathrm{it}}-u_{i}\right)\\ \;-\Lambda \left(\tau_{ik}-\mathrm{\alpha Energypovert}y_{\mathrm{it}}-\mathrm{\beta Contro}l_{\mathrm{it}}-u_{i}\right) \end{array}$$

In Equation 7, $\mathrm{Energypovert}y_{\mathrm{it}}$ represents energy poverty status, indicating whether an individual experiences energy poverty. $\mathrm{Contro}l_{\mathrm{it}}$ denotes control variables included in the model. *u_i_* captures individual effects, and *ε_it_* is the stochastic disturbance term. The parameter *α* measures the effect of energy poverty on health, while *β* represents the coefficients of the control variables. The model parameters are estimated using the maximum likelihood estimation method.

## 4 Results

### 4.1 Descriptive statistics

Overall, the mean health capital of the sample population is 2.80, indicating a moderate level of health. Approximately 41% of individuals are classified as experiencing energy poverty. The average sleep duration is 6.39 h, with a minimum of 3 h and a maximum of 11 h, highlighting significant variation in sleep patterns among respondents. Additionally, about 80% of the sample population reports having chronic diseases. The average age of the sample is 60.56 years, suggesting that the study primarily focuses on middle-aged and older adult individuals. Consumption levels exhibit considerable heterogeneity, with an average value of 6.58. Moreover, approximately 66% of the respondents engage in regular physical exercise, while 56% are male. The hospitalization rate remains relatively low, at 14% (Table 4).

**Table 4:** Descriptive statistics of variables.

Variables	Observations	Mean	Std. Dev.	Min	Max
Health	9,464	2.7959	1.0403	1	5
Energy poverty	9,464	0.4099	0.4918	0	1
Sleeping	9,464	6.3896	1.6287	3	11
Chronic	9,464	0.8036	0.3973	0	1
Age	9,464	60.5579	8.2758	45	91
Expenditure	9,464	6.5755	1.6008	3.9120	11.1152
Exercise	9,464	0.6638	0.4724	0	1
Gender	9,464	0.5647	0.4958	0	1
Hospital	9,464	0.1425	0.3496	0	1

### 4.2 Estimation results of weights

Based on the entropy method, this study analyzes the weight distribution of the energy poverty indicator system to assess the relative importance of different dimensions.

Among the primary indicators, accessibility has a weight of 31.74%. This dimension comprises two secondary indicators: electricity access and clean fuel availability, with clean fuel carrying a significantly higher weight of 96.33%, compared to electricity at 3.67%. This suggests that the availability of clean fuel is the core issue in energy accessibility, likely due to its substantial impact on health, the environment, and energy efficiency. The low weight of electricity may indicate that its accessibility is already well-established under current energy supply conditions, whereas clean fuel still requires more extensive promotion and adoption.

Convenience holds the highest weight among the primary indicators at 36.83%, underscoring the importance of ease of energy access and use. Among its secondary indicators, piped gas accounts for 86.38%, significantly outweighing tap water at 13.62%. This discrepancy suggests that accessibility challenges associated with piped gas require more focused attention than those of tap water. The complexity and high costs associated with piped gas infrastructure, coupled with geographical and economic constraints, make it a critical factor in assessing energy convenience (Table 5).

**Table 5:** Weight estimation results of MEPI.

Primary indicator	Weight	Secondary indicator	Weight
Accessibility	0.3174	Lighting	0.0367
		Cooking	0.9633
Convenience	0.3683	Piped gas	0.1362
		Water supply	0.8638
Affordability	0.3143	Share of electricity and water expenditure	0.7507
		Share of fuel expenditure	0.2492

The weight of the affordability indicator is 31.43%, closely aligning with that of accessibility. Among its secondary indicators, the proportion of water and electricity expenditure holds a weight of 75.07%, significantly higher than that of fuel expenditure at 24.92%. This distribution indicates that water and electricity costs play a more significant role in assessing energy poverty, likely because these are essential daily utilities whose price fluctuations have a more pronounced impact on household budgets. In contrast, fuel expenses constitute a smaller proportion of total energy costs and thus have a relatively lower impact.

### 4.3 The impact of energy poverty on health

#### 4.3.1 Baseline regression

Based on the baseline regression results presented in Table 6, energy poverty has a significant negative impact on health. The regression coefficient is −0.143, and the effect is statistically significant at the 5% level (*p* < 0.05). This finding indicates that, holding other variables constant, energy poverty leads to a substantial decline in health levels. This reflects that energy deprivation restricts the fulfillment of basic living needs, thereby exerting adverse effects on physical health and overall quality of life.

**Table 6:** Baseline regression results.

Variables	(1)	(2)	(3)	(4)	(5)	(6)
	health	cut1	cut2	cut3	cut4	sigma2_u
Energypoverty	−0.221***					
	(0.0522)					
Age	0.0174***					
	(0.00409)					
Gender	0.347***					
	(0.0688)					
Sleeping	0.126***					
	(0.0156)					
Chronic	−0.960***					
	(0.0702)					
Exercise	0.606***					
	(0.0487)					
Hospital	−0.839***					
	(0.0669)					
Expend	0.137***					
	(0.0217)					
Constant		−0.867***	1.474***	4.157***	5.497***	1.823***
		(0.302)	(0.305)	(0.311)	(0.315)	(0.105)
Observations	9,460	9,460	9,460	9,460	9,460	9,460
Number of ID	2,366	2,366	2,366	2,366	2,366	2,366

#### 4.3.2 Endogeneity test

When exploring the impact of energy poverty on the health capital of middle-aged and older adult individuals in rural areas, endogeneity issues may lead to biased estimates, affecting the accuracy of the results. The primary sources of endogeneity include: omitted variable bias, where unobserved variables may simultaneously influence both energy poverty and health capital, leading to estimation bias; reverse causality, where individuals with poorer health are more likely to fall into energy poverty, for example, due to reduced labor capacity resulting from poor health, which subsequently lowers income levels and affects energy access; and measurement error, where the measurement of energy poverty may contain noise, leading to imprecise variable estimation and affecting the reliability of the results. This study addresses the endogeneity issues using instrumental variables and propensity score matching methods.

This study selects housing construction materials (HCM) as an instrumental variable. Although housing conditions may be associated with health to some extent, the construction materials themselves do not directly affect residents’ health status. Instead, they influence health indirectly by affecting energy accessibility. For example, houses with brick-and-concrete structures are more suitable for the installation of clean energy facilities (such as solar water heaters and natural gas pipelines), whereas wooden or adobe houses are more likely to rely on traditional biomass fuels. Additionally, more robust housing structures typically indicate higher construction costs, which are associated with a household’s long-term economic capacity. Since economic conditions influence access to energy, housing construction materials are correlated with household energy poverty. Housing materials are typically determined at the time of construction and do not change easily in response to variations in residents’ health status. Therefore, the selection of housing construction materials can be considered relatively exogenous. This study employs housing construction materials as an instrumental variable and applies the two-stage residual inclusion (2SRI) estimation method. The 2SRI approach is an extension of the two-stage least squares (2SLS) method and is widely used to address endogeneity issues in nonlinear models (43). Specifically, in the first stage, the endogenous variable is regressed on the instrumental variable and all exogenous variables. In the second stage, the residuals from the first-stage regression are included in the model before estimation.

The results of the instrumental variable regression indicate that in the first-stage regression (Column (1) of Table 7), housing construction materials have a significant impact on energy poverty, demonstrating strong relevance. The second-stage regression (Column (2) of Table 7) shows that the negative impact of energy poverty on health capital remains significant, further confirming that the effect of energy poverty on health capital is robust and has not been substantially biased due to endogeneity issues.

**Table 7:** Endogeneity test results.

Variables	(1)	(2)	(3)	(4)
	Energy poverty	Health	Health	Health
Energypoverty		−3.258***	−0.248***	−0.258***
		(0.458)	(0.0635)	(0.0548)
Residual_Energypoverty		3.069***		
		(0.461)		
HCM	0.198***			
	(0.0209)			
Gender	−0.127	0.302***	0.398***	0.362***
	(0.0949)	(0.0692)	(0.0760)	(0.0707)
Age	−0.000187	0.0116***	0.00921**	0.0135***
	(0.00551)	(0.00414)	(0.00453)	(0.00419)
Sleeping	0.0275	0.139***	0.127***	0.123***
	(0.0214)	(0.0157)	(0.0188)	(0.0169)
Chronic	−0.00673	−0.989***	−1.016***	−1.002***
	(0.0966)	(0.0703)	(0.0833)	(0.0759)
Exercise	−0.332***	0.566***	0.602***	0.599***
	(0.0676)	(0.0491)	(0.0598)	(0.0529)
Hospital	0.104	−0.805***	−0.813***	−0.824***
	(0.0917)	(0.0672)	(0.0857)	(0.0743)
Expend	−0.842***	−0.255***	0.168***	0.175***
	(0.0328)	(0.0630)	(0.0288)	(0.0250)
Observations	9,462	9,462	5,994	7,771
Number of ID	2,366	2,366	2,257	2,360

Furthermore, the use of the propensity score matching (PSM) method helps mitigate selection bias caused by observable variables. The nearest-neighbor matching method is applied with a matching radius of 0.05, selecting the 1 and 3 nearest neighboring observations for matching. The results indicate that regardless of whether 1 or 3 neighboring observations are matched, the negative impact of energy poverty on health capital remains significant (Columns (3) and (4) of Table 7), reinforcing the robustness of the baseline regression results.

Although energy poverty may involve certain endogeneity concerns, validation through the 2SRI and PSM methods suggests that its negative effect on health capital remains consistent across different estimation approaches. Therefore, it can be concluded that endogeneity issues have not substantively affected the study’s conclusions.

#### 4.3.3 Robustness test

In empirical research, robustness analysis is a crucial step in verifying the reliability of research conclusions. This study conducts robustness checks by separately replacing the independent and dependent variables to assess the negative impact of energy poverty on health. The objective is to eliminate potential biases arising from the measurement of energy poverty and the selection of specific health dimension variables, thereby ensuring the generalizability and reliability of the findings.

To achieve this, the study replaces the independent variable with equally weighted energy poverty (EWEP, instead of using the entropy method to assign weights to each dimension of MEPI, it applies an equally weighted approach) and uses three distinct health dimensions as alternative dependent variables: mental health (Mental), daily functional health (Instrumental Activities of Daily Living, IADL5), and physical health [diseases associated with energy poverty, Asthma (44)]. Table 8 presents the regression results of the robustness tests. As shown in the table, after replacing the independent variable, the regression coefficient of EWEP for health is −0.185, which is statistically significant at the 1% level (*p* < 0.01). Additionally, energy poverty exerts a significant negative impact across all three health dimensions: in the mental health dimension (Mental), the regression coefficient of energy poverty is −0.137, which is statistically significant at the 1% level (*p* < 0.01); in the daily functional health dimension (IADL5), the coefficient is −0.223, also significant at the 1% level (*p* < 0.01); in the physical health dimension (Asthma), the regression coefficient is −0.427, with significance at the 5% level (*p* < 0.05). These findings consistently indicate that energy poverty has a substantial and statistically significant negative effect across multiple dimensions of health. The robustness analysis reinforces the reliability of the main conclusion, confirming that energy poverty adversely affects both physical and mental health.

**Table 8:** Robustness tests results.

Variables	(1)	(2)	(3)	(4)
	Health	Mental	IADL5	Asthma
Energypoverty		−0.137***	−0.223***	−0.427**
		(0.0503)	(0.0774)	(0.210)
EWEP	−0.185***			
	(0.0576)			
Gender	0.353***	0.970***	1.061***	−0.773***
	(0.0691)	(0.0754)	(0.0952)	(0.289)
Age	0.017***	−0.033***	−0.076***	−0.140***
	(0.0041)	(0.0043)	(0.0057)	(0.0169)
Sleeping	0.125***	0.220***	0.142***	0.070
	(0.0156)	(0.0154)	(0.0221)	(0.0584)
Chronic	−0.957***	−0.466***	−0.736***	
	(0.0703)	(0.0682)	(0.119)	
Exercise	0.610***	−0.088*	0.166**	−0.0977
	(0.0488)	(0.0457)	(0.0746)	(0.205)
Hospital	−0.840***	−0.307***	−0.580***	−0.736***
	(0.0670)	(0.0629)	(0.0887)	(0.222)
Expend	0.148***	0.0267	−0.00688	−0.149
	(0.0216)	(0.0205)	(0.0336)	(0.0933)
Observations	9,464	9,286	9,464	7,556
Number of ID	2,366	2,366	2,366	2,152

#### 4.3.4 Mediation effect test

According to the theoretical analysis, one of the key pathways through which energy poverty affects residents’ health is by contributing to indoor pollution. Thus, the indoor environment serves as a crucial mediating mechanism in this relationship. Households experiencing energy poverty are more likely to rely on solid fuels (such as coal and firewood) for cooking and heating (45). The combustion of these fuels not only increases air pollution but also leads to the accumulation of dust, soot, and grease indoors, thereby deteriorating the overall living environment. Additionally, energy-poor households may lack sufficient resources to maintain household hygiene, such as purchasing cleaning equipment or improving ventilation and smoke exhaust systems, further reducing indoor cleanliness. This study further investigates the mediating role of the indoor environment in the relationship between energy poverty and health capital. The regression results in Table 9 indicate a significant negative relationship between energy poverty and indoor cleanliness, suggesting that energy poverty leads to poorer indoor environmental conditions. Given that a better indoor environment has a positive impact on health, it can be concluded that part of the negative effect of energy poverty on rural residents’ health capital is realized through the deterioration of the indoor environment.

**Table 9:** Mediation effect tests results.

Variables	(1)	(2)
	Environment	Health
Energypoverty	−0.278***	−0.218***
	(0.0482)	(0.0531)
Environment		0.0925***
		(0.0228)
Gender	0.0243	0.350***
	(0.0552)	(0.0696)
Age	−0.0219***	0.0175***
	(0.00329)	(0.00413)
Sleeping	0.0314**	0.123***
	(0.0142)	(0.0159)
Chronic	−0.0759	−0.978***
	(0.0614)	(0.0714)
Exercise	0.0571	0.598***
	(0.0456)	(0.0495)
Hospital	−0.0660	−0.838***
	(0.0622)	(0.0681)
Expend	0.0944***	0.133***
	(0.0203)	(0.0221)
Observations	9,182	9,182
Number of ID	2,366	2,366

### 4.4 The heterogeneous effects of energy poverty

To further investigate the heterogeneity in the impact of energy poverty on health, this study examines how the effects vary based on education level and geographical region. And the regression results are presented in Table 10.

**Table 10:** Heterogeneous effect tests results.

Variables	(1)	(2)	(3)	(4)
	Health	Health	Health	Health
Energypoverty	−0.206***	−0.404***	−0.246***	−0.479***
	(0.0523)	(0.0977)	(0.0523)	(0.0733)
Edu	0.124***			
	(0.0221)			
Edu_energy		0.0532**		
		(0.0247)		
Region			0.393***	
			(0.0692)	
Region_energy				0.427***
				(0.0867)
Gender	0.176**	0.317***	0.361***	0.353***
	(0.0755)	(0.0706)	(0.0688)	(0.0689)
Age	0.0232***	0.0179***	0.0184***	0.0176***
	(0.0043)	(0.0041)	(0.0041)	(0.0041)
Sleeping	0.126***	0.127***	0.124***	0.126***
	(0.0156)	(0.0156)	(0.0156)	(0.0156)
Chronic	−0.958***	−0.961***	−0.955***	−0.959***
	(0.0703)	(0.0703)	(0.0701)	(0.0702)
Exercise	0.592***	0.605***	0.599***	0.608***
	(0.0488)	(0.0488)	(0.0487)	(0.0487)
Hospital	−0.837***	−0.839***	−0.836***	−0.836***
	(0.0670)	(0.0670)	(0.0669)	(0.0670)
Expend	0.129***	0.138***	0.139***	0.138***
	(0.0218)	(0.0217)	(0.0217)	(0.0217)
Observations	9,464	9,464	9,464	9,464
Number of ID	2,366	2,366	2,366	2,366

The analysis of educational heterogeneity indicates that the regression coefficient of education level (Edu) is 0.124, which is statistically significant at the 1% level (*p* < 0.01). This suggests that individuals with higher education levels tend to have better health outcomes. Furthermore, the interaction term between energy poverty and education (Edu_energy) has a coefficient of 0.0532, which is significant at the 5% level (*p* < 0.05). This finding implies that education can effectively mitigate the negative impact of energy poverty on health.

The analysis of regional heterogeneity demonstrates that the regression coefficient of the regional variable (Region) is 0.393, which is significant at the 1% level (*p* < 0.01), indicating substantial differences in individual health status across different regions. Moreover, the interaction term between region and energy poverty (Region_energy) has a coefficient of 0.427, also significant at the 1% level (*p* < 0.01), suggesting that the effect of energy poverty on health varies across regions.

## 5 Conclusion and discussion

### 5.1 Conclusion

This study analyzes the impact of energy poverty on the health capital of middle-aged and older adult rural residents in China from both theoretical and empirical perspectives. Utilizing data from the China Health and Retirement Longitudinal Study (CHARLS), the study measures energy poverty based on a multidimensional energy poverty index and examines its effect on health using an ordered Logit model. Additionally, it explores the heterogeneous effects of energy poverty by education level and regional differences. The main findings are as follows:

First, energy poverty has a significant negative impact on the health capital of rural middle-aged and older adult residents. Robustness tests reveal that energy poverty exerts significant negative effects across various dimensions of health, including mental health, physical health, and daily living capacity.

Second, individuals with lower education levels are more significantly affected by energy poverty, suggesting that higher education levels can mitigate the adverse health effects of energy poverty.

Third, the negative effect of energy poverty on rural residents’ health is more severe in southern regions compared to northern regions. This discrepancy is likely due to the lack of centralized heating in southern rural areas, where energy shortages disproportionately impact daily life, especially for the older adult in cold and damp winters.

### 5.2 Discussion

#### 5.2.1 Contributions

Our study aligns with previous research indicating that energy poverty is a major determinant of health outcomes, particularly in vulnerable populations. For instance, Kose (4) found that energy poverty in Turkey significantly worsens self-reported health outcomes. Similarly, Ma and Nie (45) explored the negative impact of energy deprivation on physical and mental health based on data from China. Our findings are consistent with their research, indicating that in rural China, energy poverty is associated with lower self-rated health (SRH) among middle-aged and older adult individuals. Furthermore, our study expands upon previous research by demonstrating that energy poverty has a heterogeneous impact across different demographic and geographic groups. While previous studies examined the adverse effects of energy poverty on health at a national level, our study reveals that individuals with lower education levels are disproportionately affected. This is consistent with the Efficient Producer Hypothesis, which suggests that individuals with higher education levels are more effective at producing health capital. Additionally, our regional analysis provides novel insights. Unlike previous studies that focused on northern China’s heating challenges, our study highlights that the adverse health effects of energy poverty are more pronounced in the south. This contradicts the common perception that colder northern regions experience the most severe energy poverty-related health risks.

#### 5.2.2 Policy recommendations

Based on our findings, we propose the following policy recommendations:

Expansion of clean energy access: Given that reliance on traditional biomass fuels significantly contributes to health deterioration, policymakers should prioritize the expansion of clean energy sources, such as natural gas and renewable energy, in rural areas. Subsidizing clean cooking fuels and improving infrastructure for energy access could help mitigate the health risks associated with energy poverty.

Education and awareness campaigns: Our findings suggest that individuals with higher education levels are better equipped to mitigate the adverse effects of energy poverty. Therefore, policies that promote education and awareness regarding energy-efficient practices and health management should be implemented. Programs that provide energy literacy training, particularly for older adults, could enhance their ability to adopt healthier energy usage behaviors.

Targeted regional policies: The regional disparities identified in our study indicate the need for region-specific energy policies. In northern China, continued investments in household heating systems and insulation could help alleviate energy poverty. In the south, where energy poverty is more strongly linked to poor health outcomes, policymakers should focus on improving access to affordable heating solutions and reducing energy costs for vulnerable populations.

#### 5.2.3 Limitations

Future research should further explore the causal mechanisms linking energy poverty and health, such as the persistence of energy poverty over time and its influence on the accumulation of health capital from a dynamic perspective. Additionally, interdisciplinary collaboration should be strengthened, integrating insights from health economics, environmental science, and social policy to examine the intersectionality between energy poverty and broader socioeconomic challenges. Such approaches will contribute to more comprehensive and scientifically informed policy designs aimed at mitigating energy poverty and its associated health risks.

## Data Availability

The original contributions presented in the study are included in the article/supplementary material, further inquiries can be directed to the corresponding author.
